# Assessment of Android Network Positioning as an Alternate Source for Robust PNT

**DOI:** 10.3390/s25237324

**Published:** 2025-12-02

**Authors:** Joohan Chun, Jacob Spagnolli, Tanner Holmes, Dennis Akos

**Affiliations:** 1 Aerospace Engineering Sciences, University of Colorado Boulder, Boulder, CO 80309, USA; jacob.spagnolli@colorado.edu (J.S.); tanner.holmes@colorado.edu (T.H.); dma@colorado.edu (D.A.); 2 Stanford University, Stanford, CA 94305, USA

**Keywords:** smartphone PNT, android network location provider, alternative PNT (APNT), network positioning, Wi-Fi fingerprinting, RFI, spoofing

## Abstract

Android devices employ several methods to calculate their position. This paper’s focus is the Network Location Provider (NLP), which leverages Wi-Fi and cell tower signals via the fingerprinting/database approach. Unlike GNSS-based positioning, the NLP should be able to compute positions even when the device is indoors or experiencing GNSS radio frequency interference (RFI), making it an enticing candidate for ensuring robust PNT solutions. However, the inner workings of NLP are largely undisclosed, remaining as a ‘black-box’ system. Using the Samsung S24 and Xiaomi Redmi K80 Ultra, we explored the NLP’s response to GNSS spoofing and offline operation (no network connection), as well as attempting NLP spoofing. The GNSS spoofing test confirmed that when satellite signals are spoofed, the NLP solution is maintained at the truth location. This reinforces the robustness of the NLP in RFI environments. In offline mode, NLP continued to operate without a Google server connection, indicating that it can compute positions locally using internally stored cache data. This behavior deviates from the conventional understanding of NLP and offers valuable insights into the latest NLP mechanism. These findings build upon previous work to uncover the inner workings of the NLP and ultimately contribute to robust smartphone PNT.

## 1. Introduction

For over a decade smartphones have been the most pervasive GNSS platform [1]. Of those smartphones, the Android platform constitutes almost 85% of the market share, making it the largest group of GNSS receivers on the planet [2]. This work will focus on Android Position, Navigation, and Timing (PNT), with a particular emphasis on robustness. While GNSS provides accurate positioning in open-sky conditions, it is highly vulnerable to radio frequency interference (RFI) and performs poorly in indoor and obstructed environments. Therefore, achieving reliable PNT on Android devices requires not only accuracy but also robustness. Various studies have been conducted to enhance the robustness of smartphone positioning. Representative approaches include spoofing and jamming detection methods that utilize the combination of C/N0 (Carrier-to-Noise Density Ratio), AGC (Automatic Gain Control), and residuals provided in NMEA (National Marine Electronics Association) messages [3,4]. In this study, we explore an alternate source of PNT available in Android devices: the Network Location Provider (NLP).

While smartphone positioning and navigation primarily utilize GNSS, they do not depend solely on satellite signals. Smartphones can incorporate various PNT sources, including leveraging a database of Wi-Fi and cell tower signals and their continually updated crowdsourced positions. These functionalities are given through three distinct Android location providers: GLP, NLP, and FLP. GLP (GPS Location Provider) utilizes signals from GNSS satellites to determine a position. NLP leverages a crowdsourced Wi-Fi and cell tower database. FLP (Fused Location Provider) combines measurements from GPS, Wi-Fi, and cell networks, as well as accelerometer, gyroscope, magnetometer and other sensors [5].

Notably, ref. [4] highlighted one of the most powerful advantages of NLP. In their experiment several cellphones were placed on a table inside a building. A spoofer placed above the phones emulated GPS L1 C/A signals to simulate a drive that began at the building and made a loop around the nearby area. The GLP and FLP positions were both misled by false satellite signals, while NLP remained unaffected and correctly maintained its original position. This suggests that NLP can play a critical role in ensuring the integrity of Android PNT. The NLP can act as a reliable back-up PNT source, which justifies its further exploration in this study.

Another previous research study assessed NLP accuracy and update rate across environments with varying network density, including indoor, rural, suburban, and urban areas [6,7,8]. The results show a strong correlation between the number of access points available and NLP performance. In high network density areas like urban areas, both positioning accuracy and update rate were higher. The update rate reached up to 0.2 Hz indoors, the highest among the tested environments. Despite having good performance in urban areas, the NLP accuracy typically remained within tens of meters, making it far worse than typical GPS performance in open-sky conditions. Nevertheless, it is available in indoor environments where GNSS signals are often unreliable or completely unavailable. Additionally, these studies compared Wi-Fi + cell positioning with cell-only positioning. Cell-only positioning resulted in NLP accuracy errors exceeding 1 km; the addition of Wi-Fi signals significantly diminished these errors and improved the solution’s accuracy.

A similar study was conducted in 2025 by Pongolini and Norlin, presenting an empirical comparison of Android’s location providers by evaluating their relative tradeoffs in accuracy and power consumption across outdoor, indoor, and vehicular environments [9]. The results of this study are comparable to the conclusions drawn by [6,7,8] in terms of accuracy, showing the GLP outperforms the NLP, and that Wi-Fi signals are a significantly more accurate and reliable than cell tower signals. This study also demonstrated the FLP’s ability to balance accuracy with power consumption by switching between location providers based on availability and required quality of service. Moayeri et al. conducted a large-scale comparison of indoor positioning accuracy for the Android FLP and Apple Core Location engine using a standardized test methodology [10]. This was done across five extensive and heavily instrumented NIST buildings. Their results showed that FLP generally achieves better horizontal and vertical accuracy than Core Location in WiFi-rich indoor environments. Their findings reinforce the importance of network positioning in indoor environments when GLP positioning is weak or unavailable.

Although some previous work has been done to analyze the accuracy, power consumption, and update rate characteristics of the NLP, its internal mechanism is still largely undisclosed and understudied, making it a black-box system. While GNSS raw measurements in Android devices are widely available and can be used to improve positioning accuracy through various post-processing methods, users only have access to the final position estimates from the NLP. Also, while Google maintains its own comprehensive database of Wi-Fi access points and cell towers, the list or map of this infrastructure is not publicly available. In order to continue to refine positioning accuracy in the android smartphone platform a more comprehensive understanding of NLP positioning and its algorithms are essential. FLP, which incorporates NLP solutions, is the location provider used by almost all applications on the android platform. This means that NLP has an impact on billions of users daily across multiple applications. In situations with degraded GLP performance, such as urban, indoor, and denied environments, this impact significantly increases. Without a robust understanding of the NLP, and its weighting in computing the FLP, the accuracy, precision, and error of the FLP cannot be definitively assessed, limiting its usefulness as a robust PNT system.

To better understand the black-box nature of the NLP, this study investigates its behavior through various empirical tests. First, a GNSS spoofing test was conducted on the latest GNSS chipsets and the latest Android version to check the status of GLP, FLP, and NLP. Reference [4] showed that NLP is robust to GNSS RFI, but this test was conducted five years ago. Since then, smartphone GNSS chipsets and the Android OS (Operating System) have seen several upgrades and updates. This effort re-proves and emphasizes that NLP can still help maintain GNSS integrity and is a reliable PNT source in an RFI environment. Subsequently, based on the findings from prior studies, this work further explores NLP processing. By conducting the test without a network connection, the operational structure of NLP is inferred. Finally, to examine the susceptibility of NLP, we conducted a limited and controlled NLP spoofing test.

Overall, this paper provides new insights into Android network positioning. A better understanding of the NLP is expected to contribute to the understanding and development of robust smartphone-based PNT solutions.

## 2. Network Positioning

### 2.1. Wi-Fi Fingerprinting

The NLP primarily utilizes the Wi-Fi fingerprinting technique [7]. Fingerprinting usually works in two phases [11,12,13]. The first is the offline training phase where signals are collected to build a radio map database. The second is the online positioning phase where received signals are sent to the database and a matching algorithm computes the user location. This technique is a representative non-ranging method that uses RSSI (Received Signal Strength Indicator). It accumulates the RSSI vectors from multiple access points in a grid-based database. The received RSSI vector from the device is then matched against the database, and the most similar RSSI pattern is selected to estimate the user’s location. One of the key advantages of RSSI-based fingerprinting is that it does not require additional infrastructure. Wi-Fi signals are already pervasive in our surroundings. Moreover, RSSI can be received even if the Wi-Fi access point is not connected to the internet. However, building a database over a wide area requires significant time and effort.

Google went into greater detail how it builds and utilizes its network database in a letter released to the Defense Priorities and Allocations System [14]. Google constructs a large-scale access point database similar to typical fingerprinting methods, but the positioning approach is fundamentally different from it. Although NLP still uses RSSI, the location is estimated using an RSSI-based ranging technique.

**Figure 1:**
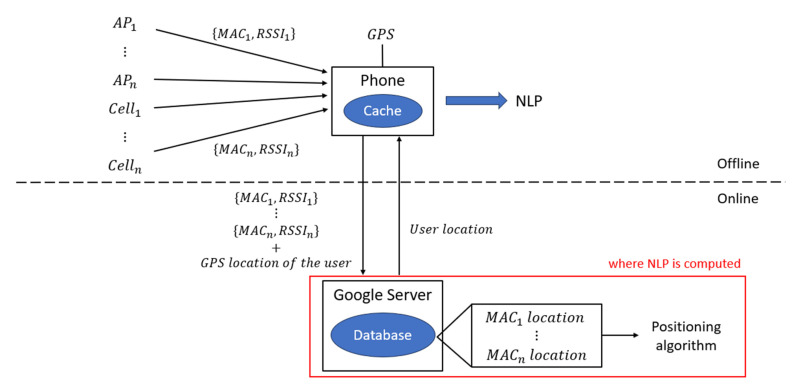
NLP diagram. The server computes NLP and sends it back to users. NLP operation may require a network connection.

Specifically, Google tracks the media access control (MAC) address of the access point and the GPS location of the user’s device at the point at which the access point was visible to build its database. Google services can then use this large collection of Wi-Fi access point data as follows. The user’s device sends a request to the Google location server with a list of MAC addresses which are currently visible to the device. The server compares the visible MAC addresses with the stored MAC addresses in the pre-built fingerprint database to identify the associated geocoded location. Based on this, the server calculates the user’s distance from each access point as a function of received signal strength and uses this to triangulate the approximate location of the user. The approximate location is sent back to the device as the NLP output. This is illustrated in Figure 1.

One of the main advantages of traditional fingerprinting is that it does not rely on ranging, making it less vulnerable to distance errors caused by non-line-of-sight (NLOS) conditions. For this reason, it can perform well even in indoor environments with obstacles. In contrast, NLP is based on RSSI-based ranging, therefore requires more precise and accurate distance measurement to enhance location accuracy. To address this limitation, Android introduced Wi-Fi RTT (Round-Trip-Time), a technology that measures distance based on the time-of-flight (TOF) of radio signals, in 2018 [15]. It measures the time taken for a Wi-Fi RF packet to travel from an access point to the phone and back. Since radio signals travel at the speed of light, multiplying the round-trip time by the speed of light and dividing by two yields the distance between the phone and the access point. This technique has been reported to achieve accuracies of 1–2 m [16]. However, hardware limitations exist. Both the smartphone and the access point must support the IEEE 802.11-2016 FTM (Fine-Time-Measurement) standard [17]. Wi-Fi RTT technology has been mentioned as being integrated into FLP to support more accurate indoor positioning [15].

### 2.2. Cellular Positioning

Although it is not as accurate as Wi-Fi fingerprinting, the NLP in Android also utilizes cell tower signals [18]. Traditional cellular positioning methods include Time Difference of Arrival (TDOA), Angle of Arrival (AOA), and Time of Arrival (TOA), which rely on precise temporal or angular measurements between the device and multiple cell towers. These methods typically require tight time synchronization between transmitters and receivers, and are fundamentally limited by the relative sparse deployment density of cell towers compared to Wi-Fi access points. However, the emergence of high-frequency, wideband 5G signals holds the potential to improve positioning accuracy by enabling finer time resolution. Despite this, NLP does not leverage advanced techniques like TDOA or AOA. Instead, similar to Wi-Fi fingerprinting, it relies on cell-ID and received signal strength (RSSI), referencing a pre-built database of cell tower locations to estimate the user’s position [19].

## 3. Test Scenario

**Figure 2:**
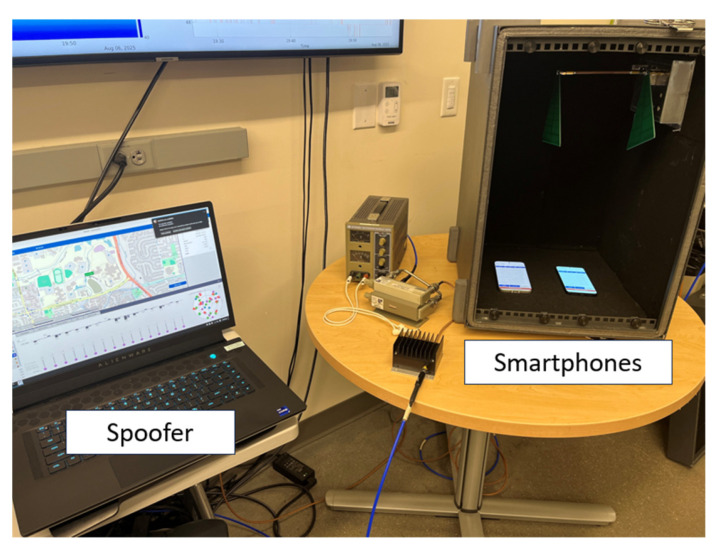
Setup of spoofer and smartphones. The recording devices remain stationary inside the chamber throughout the experiment.

The experiments are divided into two categories: a GNSS spoofing test, and an offline NLP test. The GNSS spoofing test was conducted at the Ann and H.J. Smead Aerospace Engineering Building of the University of Colorado Boulder. The smartphones used for testing were devices with the latest GNSS chipsets. A list of them is provided in Table 1. The devices were placed inside a test chamber, as illustrated in Figure 2. Despite being in the chamber, they maintained access to Wi-Fi and were able to obtain NLP solutions. Live GNSS signals received via a Novatel Pinwheel antenna installed on the roof of the building were also rebroadcast into the chamber. The devices were allowed to compute the true rooftop position for five minutes before spoofing signals generated by the SkyDel SDX Software-Defined GNSS Simulator (Orolia, Montréal, QC, Canada) were introduced. The spoofing signals were designed to simulate a route starting from the building and traversing around the surrounding area. The test was carried out under three scenarios: the first involved only the GPS L1 C/A signal, while the second included high-band signals from all available constellations—GPS L1, Galileo E1, GLONASS G1, and BeiDou B1. The third scenario extended this to include both high-band and low-band signals from all constellations.

Another set of tests were designed in offline mode to better understand the NLP architecture. The published architecture (Figure 1), where the NLP is computed on the server, implies that a network connection is essential for NLP to function. However, it was observed in a preliminary test that NLP updates are still available while walking inside a campus building without a SIM card, nor a Wi-Fi connection. This clearly indicates that NLP does not require network connectivity, and therefore, the published architecture cannot be correct. This observation challenges the conventional understanding of NLP as a purely server-based service in which the user’s location is determined on Google’s server and then transmitted back to the device. Instead, it suggests the presence of an alternative mechanism by which the NLP can operate locally on the device using cached data.

Thus in addition to the GNSS spoofing test, an experiment was constructed to further assess and understand the network dependence for NLP.

Two round-trip drives from Boulder to Denver, CO were conducted for the cache test. For the first run, the devices were online with both a SIM card and Wi-Fi enabled. During this phase, it was assumed that the phone continuously communicated with Google servers, collecting and caching access point data along the route. For the second run, the devices were offline with their SIM card disabled and Wi-Fi enabled but disconnected. This ensured the device would not be able to communicate with any Google servers or databases but could still scan for Wi-Fi signals. Both drives initially followed the same route but the second continued further south through Denver to previously unvisited regions and then returned to the original path that the first drive followed during the online phase. To ensure that the offline NLP performance relied solely on the cache built during the first online drive, the smartphone was factory reset immediately prior to this test. Note that all devices were equipped with an active SIM card, ensuring strong cellular coverage from a major provider and guaranteeing identical coverage across the phones.

Google’s GnssLogger App was used to collect GLP, FLP, and NLP logs. The status of Wi-Fi and cell tower connectivity was recorded using the WiGLE WiFi Wardriving App. It provides log data such as MAC, SSID, RSSI, frequency, and signal type (e.g., Wi-Fi, GSM, WCDMA, LTE). Although access to Google’s network database is restricted, the WiGLE App allows researchers to determine the number of APs and cell towers detected and to analyze how changes in these counts impact NLP. A summary of all the tests is provided in Table 2.

## 4. Results

### 4.1. GNSS Spoofing Test

**Figure 3:**
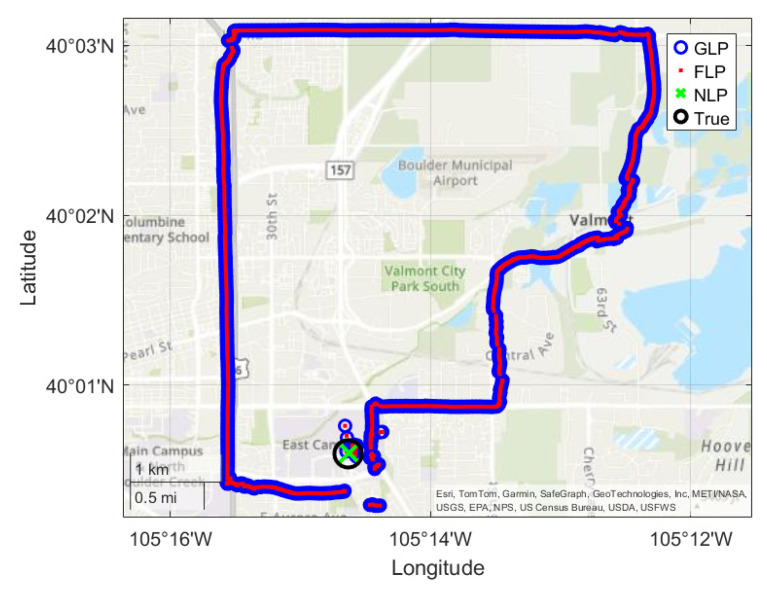
Redmi K80 Ultra result from spoofing experiment. The GLP and FLP were successfully spoofed. Although FLP incorperates network sources, the spoofing of GNSS receivers was sufficient to manipulate FLP. NLP was not affected by spoofing and remained its original location.

For all the spoofing scenarios, NLP correctly reported its location in the aerospace engineering building. However, the behavior of GLP differed from that observed in a previous study [4]. When only GPS L1 C/A signal was spoofed, the phones used GPS L5 and other constellation signals to maintain their position. This result suggests that the robustness of GNSS receivers has significantly improved compared to five years ago. In the past, even when L1 and L5 signals from all constellations were being tracked, the phones would follow the spoofed GPS L1 C/A signal [4]. This result shows that phones are now able to use other signals to compute an accurate position in the presence of GPS L1 C/A spoofing. Even when all high-band signals were spoofed, both the S24 and Redmi K80 Ultra used GPS L5, Galileo E5a, and Beidou B2a signals and did not follow the spoofed position. Previously, phones were unable to continue using only L5 signals under L1 high-band RFI conditions and were consequently spoofed. However, phones are now able to continue tracking the previously acquired L5 signals in the presence of jamming or spoofing of the L1 band and can maintain the correct position. Nevertheless, a structural dependency still remains: the phones must first acquire the L1 signals and rely on them to initiate L5 signal acquisition. This underscores that dual-frequency support enhances not only the accuracy but also the robustness of GNSS receivers, providing redundancy and resilience under GNSS RFI conditions. Finally, all phones were immediately spoofed when all signals were included (both low- and high-band signals), while the correct network positioning is still available as shown in Figure 3.

FLP followed the GLP position in all scenarios. Notably, even when all signals were spoofed and GLP reported an incorrect position, FLP appeared to rely more on GLP than on NLP—despite NLP maintaining the correct location. This behavior reaffirms that, in GNSS RFI environments, NLP can be a more reliable source than both GLP and FLP.

The statistical results of these tests can be found in Table 3 below. To calculate these errors, the mean solutions of FLP and NLP were computed prior to the introduction of the spoofed signals for each test, representing the nominal locations under standard conditions. Then, the solutions of each after the introduction of the spoofed signals were compared to these true locations, generating the error metrics. While the nominal locations the errors are being compared to do not exactly represent the true location of the devices, this comparison allows for easy calculation and visualization of the effect that spoofing has on the position solution.

Notably, in the case of the Samsung S24, there are small numbers of NLP solutions that differ by more than a kilometer. This is due to the NLP solution jumping nearer to a cell tower due to the limited number of Wi-Fi signals accessible to the phone. These solutions do not last long, and nominally the NLP solution is in the correct location. A similar phenomenon was observed in the Redmi K80 Ultra when all high-band signals were spoofed, but for a longer period and further away, significantly effecting the mean error values. Also, when exposed to all high-band signals, the Redmi K80 FLP solution was spoofed briefly, resulting in large max error values in FLP, but was nominally in the correct location.

**Figure 4:**
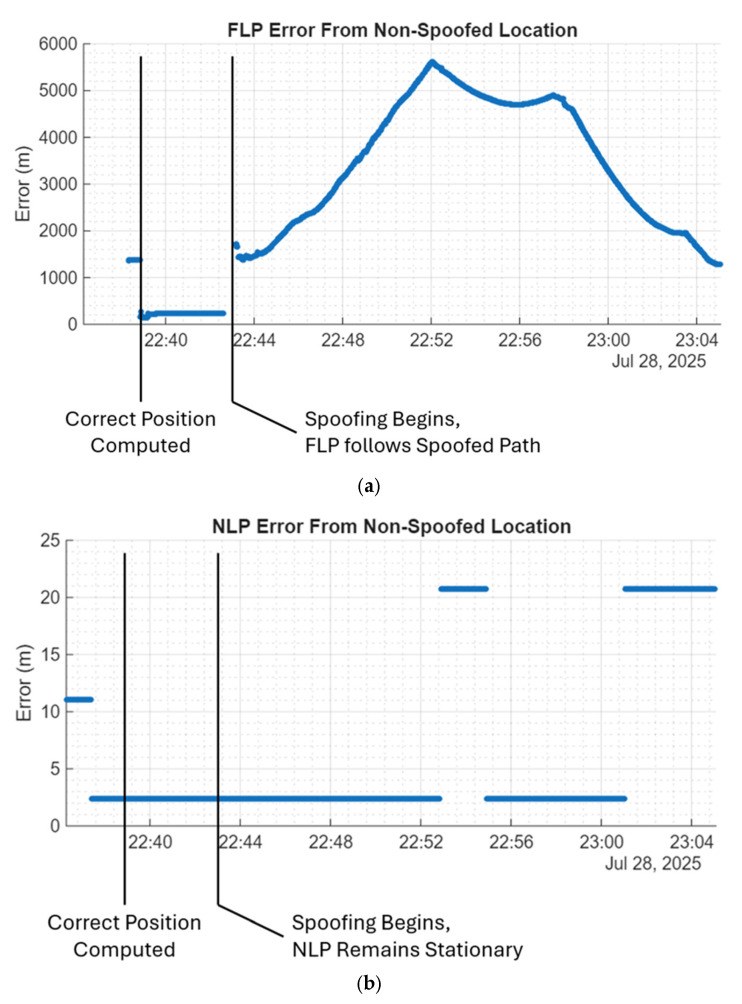
(**a**) Redmi K80 Ultra result from all signal spoofing experiment. The blue line in this graph shows the 3D RMS error in FLP position compared to the non-spoofed location. The FLP position follows the spoofed path soon after spoofing begins. (**b**) Redmi K80 Ultra result from all signal spoofing experiment. The blue line in this graph shows the 3D RMS error in NLP position compared to the non-spoofed location. The NLP position remains mostly stationary even after spoofing occurs.

In addition, the error values when all signals are being spoofed are plotted by coordinate for both the FLP and NLP solutions for the Redmi K80 Ultra in Figure 4a,b respectively. Since the phones were not spoofed in either the GPS L1 C/A only test or the all high-band signal test, these plots are not enlightening and thus are not included. The included plots help illustrate how the NLP solutions remain stationary in the presence of spoofing, while the FLP follows the spoofed path. This shows how in the presence of spoofing, NLP can be a more reliable source than FLP.

### 4.2. Offline Test

**Figure 5:**
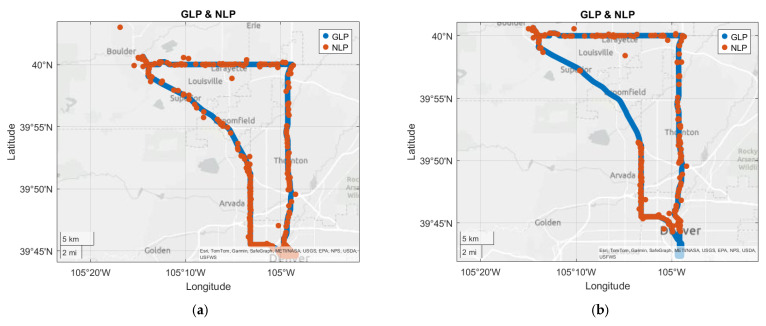
Test route and NLP results from Samsung S24. (**a**) is online, (**b**) is offline. NLP updates are available even without network connection. However, they stop when the user moves to South Denver, a region that was not visited during the previous online session, and resume as the user approaches the online session drive path again.

In online mode, NLP updates were consistently generated along the entire route at an update rate of approximately 0.08 Hz, as illustrated in Figure 5a. In offline mode, NLP updates were also generated at a similar rate and accuracy, matching the performance observed during the online test. However, upon continuing to south Denver—a region not covered during the online session—NLP updates ceased completely. As the vehicle returned toward downtown Denver, NLP updates resumed at a specific point before fully reentering downtown. From that point onward, NLP continued to function normally enroute to Boulder. Figure 5b shows the offline test results. One point to note is that on the highway section from Boulder to Denver, the NLP update rate was significantly lower compared to the online case; this is further discussed in Section 4.2.3.

These results confirm two important findings: First, on-device NLP computation is possible. Second, on-device NLP depends heavily on cached data. The cached entries are presumably obtained from the server during prior online operation. When such cache entries were unavailable, NLP updates did not occur—even if Wi-Fi signals were being received—indicating that uncached APs are ignored in offline mode.

#### 4.2.1. NLP Architecture

**Figure 6:**
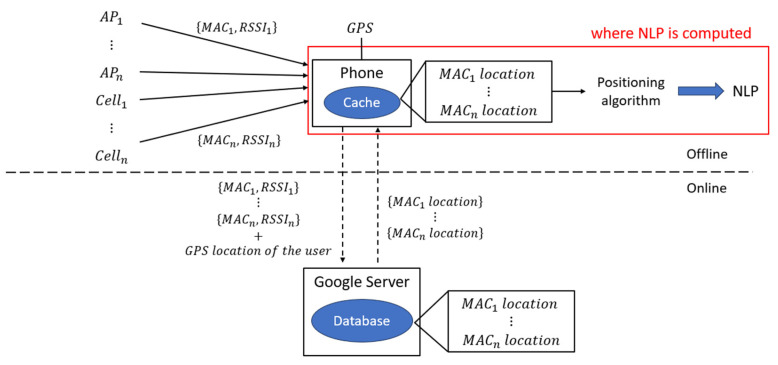
The latest inferred NLP diagram. In offline mode, the device computes NLP positions based on locally cached data.

Based on the cache test results, a different NLP architecture can be inferred. According to Google’s previously disclosed architecture (Figure 1), NLP operates by sending the list of nearby access point MAC addresses and RSSI to the server, which in turn returns a computed user location. However, as illustrated in Figure 6, the device itself appears capable of performing the location estimation. Also, the data exchanged with the server may no longer be the final user location, but rather the location of individual APs, which the device can store and utilize independently. It remains unclear what kind of data is exactly exchanged between the phone and the server. For instance, if NLP computation is performed on the server, the RSSI values must be transmitted from the phone to the server. That is because the NLP fingerprinting method calculates the distance as a function of signal strength as discussed in Section 2.1. However, if the computation occurs on the device, these values may be used locally without being sent.

#### 4.2.2. Cache Coverage

The fact that NLP operates in an offline state indicates that it can be used even in environments with limited network connectivity. This can be considered another advantage of NLP. At the same time, the reliance on cache data received from the server functions as a constraint. This section of the study examined how far this constraint, or the effective coverage of the cached data, might extend.

**Figure 7:**
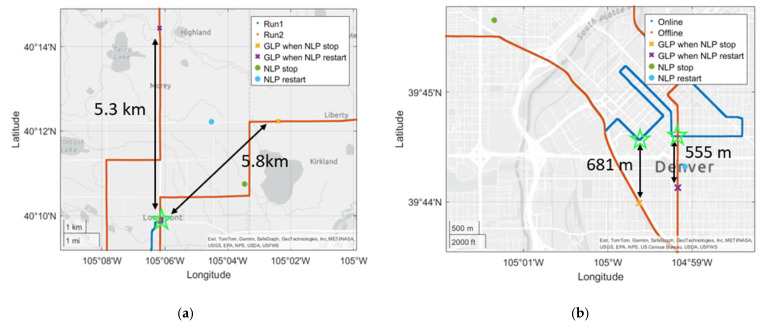
The horizontal distances of two GLPs when NLP stopped and resumed from the nearest GLP locations (green star marker) during the online phase. (**a**) is a suburban/rural test conducted between Boulder and Longmont, CO using the Samsung S24. The average of two distances of approximately 5.5 km implies the estimated cache coverage in a suburban/rural area. (**b**) is from the same urban test as in Figure 5 above, also using the Samsung S24. The average of two distances of approximately 618 m implies the estimated cache coverage in an urban area.

To estimate the spatial coverage of the cached data, the GLP positions at which NLP updates stopped and resumed during the offline test were compared to the nearest GLP positions observed during online tests. Figure 7a shows a suburban/rural route while Figure 7b shows the urban route. These two routes were chosen as they had different density of Wi-Fi access points. Figure 7a shows that the NLP updates ceased at 5.8 km from the closest GLP point recorded and resumed 5.3 km away during the return trip. For the urban route these values were 555 m and 681 m, respectively. By taking the average of the two distances from each test, it can be inferred that the effective coverage radius of the cached data was approximately 5.5 km for the suburban route and 618 m for the urban route.

Although the propagation and reception range of Wi-Fi signals is typically limited to just a few tens of meters, the fact that NLP updates were still generated at locations hundreds of meters away from the furthest point reached during the online tests suggest that the cache includes more than just the access point information directly received by the phone. It can be inferred that, based on the GPS location during the online phase, the server also pushed information about nearby Wi-Fi access points that the phone did not directly observe, and this information was stored as cache on the device.

These results also confirm that the effective range in which offline NLP operates varies depending on the number of Wi-Fi access points in the area. Since the size of access point data pushes from the Google server are likely to be fixed, the effective radius of cached data in dense urban environments is likely smaller due to the higher number of access points. It is likely there is a direct relationship between access point density and cache coverage radius, but further testing in differing environments would be required to prove this. This has implications for offline NLP operation, especially considering it is more likely to be useful in the dense urban environments where GLP may not be available.

#### 4.2.3. Horizontal Accuracy

The Android network location engine provides not only the NLP position but also an expected accuracy value. According to Google, this expected accuracy represents a radius with 68% confidence [20]. In this study, the actual error was defined as the horizontal distance between the NLP and GLP positions, and this was compared against the Android estimated accuracy. In the online mode, 62.50% of the NLP errors were smaller than the Android estimate, and in the offline mode, 66.05% were within the estimated bounds. These results suggest that the Android report accuracy estimates can be considered reasonably reliable in both online and offline conditions.

**Figure 8:**
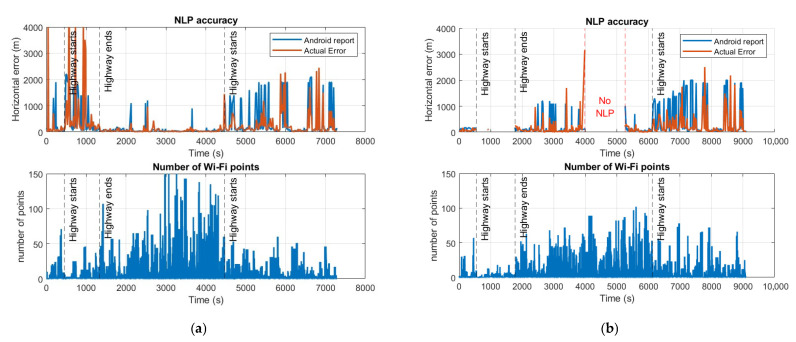
Horizontal accuracy of the NLP versus GLP and Android report 68% CEP. (**a**) illustrates the results from the online mode. (**b**) represents the results from the offline mode. These are results from the Samsung S24, using the same dataset as Figure 5 above.

The offline test involved driving through two significantly different environments: a highway and downtown Denver. As expected, the accuracy varied considerably between these environments. On the highway, the mean error was relatively large at 185.21 m, while in suburban and urban areas, the mean error was lower at 95.89 m. Furthermore, the variation in NLP accuracy showed a clear correlation with the number of Wi-Fi access points received during each segment as shown in Figure 8.

One notable observation is that during the highway segment in offline mode, NLP updates were rarely generated. This appears to be due not only to the limited number of Wi-Fi access points detected in that segment, but also to the high-speed movement, which caused frequent signal acquisition and loss. As a result, the number of available signals may have been insufficient for NLP computation. Further investigation is needed to assess the impact of user dynamics on NLP performance.

#### 4.2.4. Device-Specific NLP Variations

**Figure 9:**
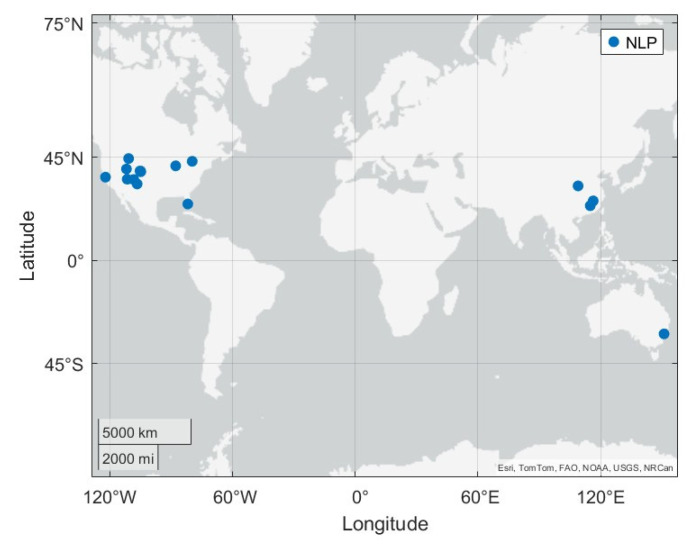
Online mode operation of the Redmi K80 running the China ROM. This plot demonstrates the abnormal NLP results produced by the phone while operating dynamically in the Boulder and Denver, CO area. No NLP plot is available for the Redmi K80 in offline mode operation as no position solutions were able to be generated.

According to the Google open-source framework repository, the location provider is only able to be modified at the OEM (Original Equipment Manufacturer) level, and modifications or updates by third parties or individual developers are restricted [21,22]. Most manufacturers use the NLP and FLP offered by Google Mobile Services (GMS), which means that NLP operates based on the same algorithm and database regardless of the device manufacturer. In other words, NLP does not depend on the manufacturer. In contrast, Android smartphones with a China ROM (Read-Only Memory) cannot use GMS and must implement their own NLP and FLP algorithms. As a result, there was a significant difference in NLP performance between the Non-GMS Xiaomi Redmi K80 Ultra, which is based on a China ROM, and the GMS-based Samsung S24. Figure 9 shows the NLP results from the Redmi K80 during an online drive test. This data was collected on the same drive that was shown in Figure 5a for the Samsung S24. Despite the test being conducted in Boulder and Denver, CO, the Redmi K80 produced abnormal NLP positions, reporting locations in the eastern United States and even in China. Furthermore, in offline mode, NLP did not function at all on the device, never producing a position solution.

## 5. Potential Vulnerability of NLP

While NLP enhances smartphone PNT robustness under GNSS RFI in both online and offline mode, it is important to explore if it too has vulnerabilities. To investigate the susceptibility of NLP, we conducted a limited and controlled test. As expected, no system is entirely infallible and even NLP can be “tricked”.

**Figure 10:**
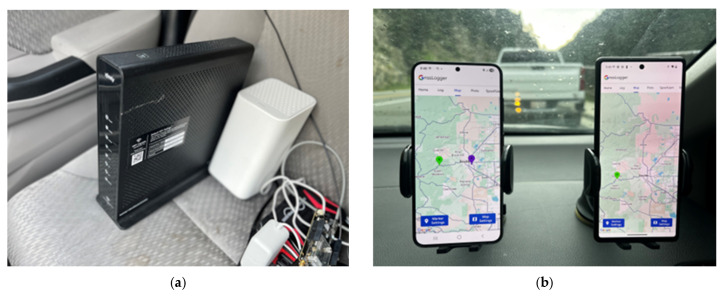
Setup of (**a**) routers and (**b**) smartphones. Home Wi-Fi routers were brought to the canyon with the phone inside a vehicle and powered on.

In this test, smartphones and home Wi-Fi routers were brought to a canyon in Boulder, CO. Figure 10 represents how the phones and routers were placed in the vehicle. The routers had been stationary at known locations for 10 months prior to the test, so they were expected to have their locations registered in Google’s network database. This allowed the relocated Wi-Fi router to act as a false signal to the test phones. The canyon location was specifically chosen to eliminate interference from other Wi-Fi or cell tower signals, ensuring that the relocated router is the only dominant network signal available for NLP computation. Moreover, since the canyon had no cellular service, the experiment was performed in an offline state. The smartphones’ cache data was controlled by first factory resetting the devices to clear all previously cached data, then placing them near the routers of interest in their original locations while connected to the network to allow the newly empty cache to download the routers’ GMS database location. The original locations of the two routers were taken via GPS survey before being placed in the vehicle and driven to the canyon to conduct the test. It is important to note that these surveyed positions are unlikely to be identical to the geocoded location of the access points used by the NLP in the Google database. This discrepancy would be due to the fingerprinting method the database uses to encode locations. However, because the access points were stationary for a long duration of time in high traffic locations the difference between the survey and database location is likely minimal.

**Figure 11:**
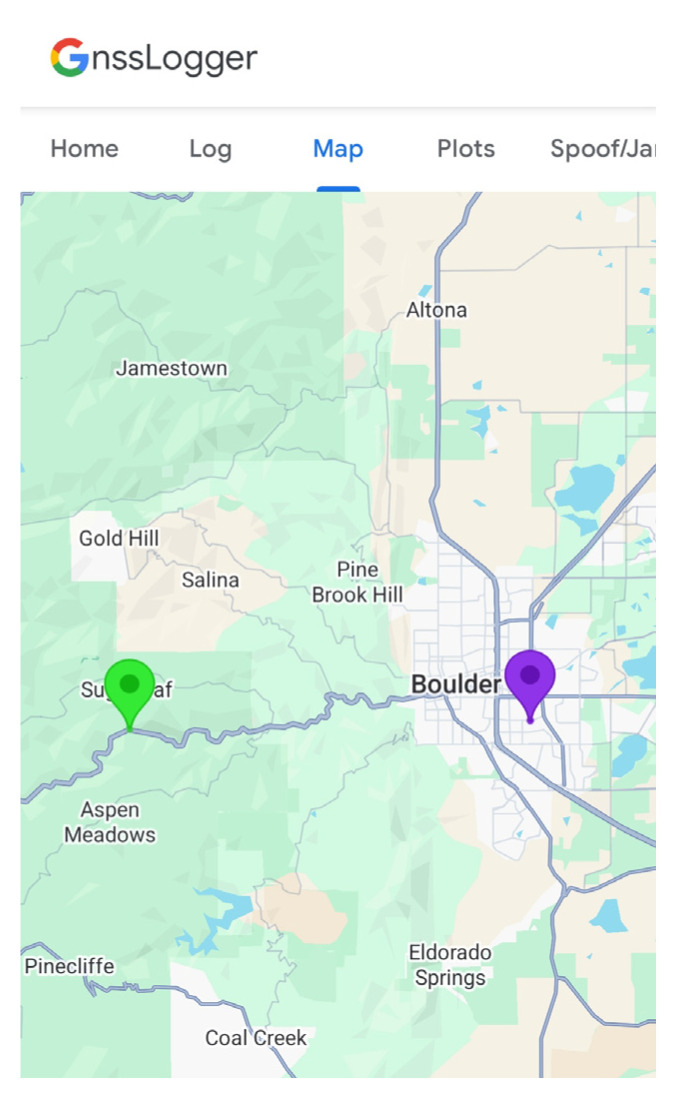
Samsung S24 result from NLP spoofing experiment. NLP was successfully spoofed to the original location of the Wi-Fi access point. The green marker on the left indicates the actual location of the device, based on GLP, while the purple marker on the right represents the NLP. The actual location of the device and the location of the spoofed NLP were approximately 12 km apart.

As soon as the routers were powered on in the canyon and the phone received the signals, NLP was successfully spoofed. By using the reported latitude and longitude of the spoofed NLP position from the S24 and the original locations of the Wi-Fi routers, converting to north and east positions using the UTM (Universal Transverse Mercator) coordinate system, and taking the difference, error values were computed. These values are listed below in Table 4. These results show that the spoofed location is very close to the original location of one of the two access points: Router 1. The error values calculated for Router 1 are within the expected precision for NLP solutions, leading to the conclusion that the spoofed NLP position is the original location of Router 1. The reason the spoofed position was at the original location of one of the two access points is ambiguous, but potential factors could include the time for the routers to turn on and connect to the device and their received signal strength. Router 1 had an edge in both cases, as the S24 saw Router 1 first, and the received signal strength of Router 1 was 6.5 dB higher than that of Router 2. Figure 11 shows the GnssLogger App used for NLP logging. It clearly illustrates how NLP (purple marker) was spoofed to the original router location, resulting in a position estimate entirely different from that of GLP (green marker). Notably, even though Android reported an NLP accuracy of 20 m at the time, the actual horizontal distance between the NLP and GLP positions was approximately 12 km. Although NLP previously demonstrated robust performance under GNSS RFI, it was notably deceived in this experiment. A simple relocation of a home Wi-Fi router—an action easily carried out by any user—was sufficient to manipulate the NLP output.

One additional observation made during this testing was that NLP seemed to require a minimum of two Wi-Fi access points to generate a location estimate. In the canyon test, no NLP solution was available when only one access point was used, whereas the presence of two access points enabled successful position estimation and spoofing.

This result does not contradict the robust nature of NLP, but rather highlights that, as with any system, vulnerabilities exist. In practice, most users maintain continuous cellular connection and are in view of a wealth of Wi-Fi access points, making NLP spoofing much less of a concern than its GNSS counterpart. Nonetheless, with electronic warfare and RFI on the rise, bringing attention to a potential vulnerability early could be paramount in keeping it from becoming a more pervasive issue in the future.

## 6. Conclusions

This study evaluated the performance of the Network Location Provider (NLP) in Android smartphones across a variety of real-world environments and analyzed key factors influencing its performance. First, we reconfirm its role as an alternative PNT source under GNSS RFI conditions, where both GLP and FLP were misled by false signals, while the NLP remained unaffected. Second, NLP updates were observed even in offline mode, suggesting that devices compute NLP positions based on locally cached data. This behavior deviates from the conventional understanding of NLP as a purely server-based service and offers valuable insights into the latest evolution of NLP mechanism. Our results further suggest that the spatial coverage of offline NLP depends on the density of previously observed Wi-Fi access points: in urban areas with dense networks, cache coverage tends to be smaller than in suburban areas. In a controlled test, relocating a stationary Wi-Fi router successfully spoofed the NLP position to the router’s original location, revealing a potential vulnerability. However, because this experiment was conducted under highly restricted conditions in which only the router’s signal was received, such spoofing is expected to be extremely difficult in typical user environments. Thus, despite this limitation, NLP can still serve as a valuable APNT source, enhancing the robustness of GNSS-based positioning. Finally, although NLP can function without a network connection, its spatial validity is limited and may be unreliable if Wi-Fi infrastructure changes; maintaining a continuous network connection is therefore recommended for reliable NLP operation.

Future work could investigate additional factors affecting NLP performance. NLP algorithm modification is limited to OEM-level implementations through Google Mobile Services (GMS). Unless a manufacturer customizes their own NLP logic, most Android devices share the same underlying algorithm and database. This implies that NLP is more dependent on software architecture than on specific hardware models. However, differences in hardware—such as the number of detectable signals, RSSI sensitivity, and cache memory capacity—may still influence NLP performance across devices. Further analysis also remains to be done on what factors contribute to the weighting of the GLP and NLP solutions when computing the FLP. The spoofing tests in Section 4.1 demonstrate the FLP directly following the GLP in a spoofed scenario, but further research may be conducted to break down when and how the FLP utilizes the NLP, especially in indoor environments.

The techniques utilized in this work for android devices may also be useful in deciphering the operation of other NLP-like engines such as those used by Apple smartphones. Apple states they utilize information from cellular, Wi-Fi, GPS, and Bluetooth to determine your location [23]. Additionally, they explicitly state “iOS and iPadOS devices without a cellular connection use only Wi-Fi for Location Services” [23]. The difficulty in working with the iOS platform is the lack of positioning information output by the device, as they only output the final position. The APIs provided by the Android platform allow GLP and NLP positions to be reported separately, greatly enabling the ability to perform the analyses in this work.

Wi-Fi RTT has been introduced as a more precise ranging-based positioning method, but current implementations appear to be integrated within the FLP. While combining multiple sources through FLP can improve accuracy, our findings show that FLP tends to follow GLP under GNSS RFI, which reduces its robustness. In contrast, NLP—which is used as an independent location provider—demonstrated resilience in such scenarios. Therefore, continued research into the behavior and limitations of NLP is essential. Deeper understanding of its dependencies and vulnerabilities will contribute valuable insights to the field of Alternative PNT.

## Figures and Tables

**Table 1:** List of the test devices and the GNSS chipsets inside them.

Device	GNSS Chipset
Redmi K80 Ultra (Xiaomi, Beijing, China)	MediaTek Dimensity 9400+
Galaxy S24 (Samsung, Seoul, Republic of Korea)	Qualcomm Snapdragon 8 Elite

**Table 2:** Overview of all the tests investigated in this study.

Scenario	Details	Test Region
GNSS spoofing	Spoofing GPS L1 C/A only, high-band signals, then all signals	Ann and H.J. Smead Aerospace Engineering Building
Offline	Driving test with and without network connection	Denver, CO

**Table 3:** Numerical error results from GNSS Spoofing Test.

	Samsung S24				Redmi K80			
Spoofing Scheme	FLP		NLP		FLP		NLP	
	Mean (m)	Max (m)	Mean (m)	Max (m)	Mean (m)	Max (m)	Mean (m)	Max (m)
L1 C/A Only	7.62	22.93	41.93	1591.1	2.58	43.79	6.12	23.80
High-Band	25.62	30.14	7.74	1623.1	83.42	3180.4	526.86	3851.9
All Signals	1852.2	7172.4	28.71	1680.8	2715.8	5621.2	3.78	20.71

**Table 4:** NLP Spoofing Test Results.

	North Error (m)	East Error (m)	Range Error (m)
Router 1	13	−29	31
Router 2	−1603	641	1726

## Data Availability

The original data presented in the study are openly available in Zenodo repository at https://doi.org/10.5281/zenodo.16995538.
